# Nonpharmaceutical Influenza Mitigation Strategies, US Communities, 1918–1920 Pandemic^1^

**DOI:** 10.3201/eid1212.060506

**Published:** 2006-12

**Authors:** Howard Markel, Alexandra M. Stern, J. Alexander Navarro, Joseph R. Michalsen, Arnold S. Monto, Cleto DiGiovanni

**Affiliations:** * The University of Michigan Medical School, Ann Arbor, Michigan, USA,; † The University of Michigan School of Public Health, Ann Arbor, Michigan, USA;; ‡ US Department of Defense, Fort Belvoir, Virginia, USA

**Keywords:** 1918–1920 pandemic influenza, nonpharmaceutical interventions, protective sequestration, quarantine, isolation, pandemic mitigation or containment strategies, dispatch

## Abstract

We studied nonpharmaceutical interventions used to mitigate the second, and most deadly, wave of the 1918–1920 influenza pandemic in the United States. We conclude that several small communities implemented potentially successful attempts at preventing the introduction of influenza.

The 1918–1920 influenza pandemic was the deadliest pandemic in human history (*1**–**6*). We undertook a historical evaluation of nonpharmaceutical interventions (NPIs) during that pandemic (*7*), with an emphasis on American communities during the second wave (September–December 1918). The full report and a digital archive of primary sources for this study can be accessed online (available from http://www.med.umich.edu/medschool/chm/influenza).

## The Study

We selected 6 US communities that reported relatively few, if any, cases of influenza and no more than 1 influenza-related death while NPIs were enforced during the second wave of the 1918 pandemic: San Francisco Naval Training Station, Yerba Buena Island, California; Gunnison, Colorado; Princeton University, Princeton, New Jersey; Western Pennsylvania Institution for the Blind, Pittsburgh, Pennsylvania; Trudeau Tuberculosis Sanatorium, Saranac Lake, New York; and Fletcher, Vermont (Table). We also studied the college community of Bryn Mawr College, Bryn Mawr, Pennsylvania, which took several intensive NPI measures and experienced no deaths during the second wave but did encounter a high case rate (25% of its student body). We identified these sites first by consulting Jordan's 1927 text, Epidemic Influenza (*1*). We then verified and modified this list by reviewing 240 federal, 92 state (from 40 states), and 25 special local reports and documents. We conducted in situ archival research at 34 locations and examined >1,400 newspaper and contemporary medical and scientific journal articles for the 1918–1920 period.

**Table:** Six communities that along with Bryn Mawr College escaped influenza pandemic, 1918–1920

Characteristics	Yerba Buena, CA	Gunnison, CO	Princeton University, NJ	WPIB, PA*	Trudeau Sanatorium, NY	Fletcher, VT	Bryn Mawr College, PA
Population	≈6,000	1,329 in town, 5,590 in county	1,142	179 students; faculty and staff also lived on-site	356 patients admitted in 1918; 259 discharged; average daily patient census of 150	737	465
Geographic isolation	Small island off coast of San Francisco	Small mountain community in western Colorado; commercial, educational, and transportation hub	Student body in a small college town; campus somewhat separated from the town	Located in a busy residential Pittsburgh neighborhood but somewhat isolated by standards of the day	A small institute on the outskirts of a very small mountain community in upstate New York	Very small rural community in upstate Vermont	Student body in a small college town; only 10 miles from Philadelphia
Ordinary or special population	Primarily a military population; ≈1,000 civilian family members and workers were present	Ordinary population composed of native-born and immigrant residents	All-male student body; 92% of students were members of a military training corps	Student body was blind and thus isolated by standards of the day	Patients and staff were tubercular and were thus isolated by standards of the day	Ordinary rural population	All-female student body
Period of protective sequestration	Sep 23 – Nov 21,1918	Oct 31, 1918 – Jan 20, 1919 (countywide); public closures and imposed social distancing as of Oct 8, 1918	Never under a full protective sequestration, as recruits and cadets continually arrived and left; restrictions on off-campus travel (with perimeter control) imposed Oct 8–Dec 21, 1918	Early Oct – late Nov 1918	A de facto protective sequestration existed due to its geographic and institutional isolation	Not applicable	≈Oct 1–Nov 7, 1918
Cases and deaths	0 cases, 0 deaths during protective sequestration	0 cases, 0 deaths in town (2 cases, 1 death in county)	68 cases, 0 deaths in student population†	12 cases, 0 deaths	0 cases, 0 deaths	2 cases, 0 deaths	110 cases, 0 deaths

*WPIB, Western Pennsylvania Institution for the Blind. †One professor died.

The communities we identified were diverse and had unique characteristics. Fletcher, Vermont (population 737), was simply too small to suggest that its success resulted from anything more than remote location, good fortune, or the ways in which the virus skipped some communities altogether for unknown reasons (*8**–**10*). The Trudeau Tuberculosis Sanitarium (*9*) and the Western Pennsylvania Institution for the Blind (*10*) were already de facto quarantine islands because of the era's prevailing views toward confinement of the contagious and the disabled. Princeton University provided a good example of how a social institution with some measure of control over its population might implement NPIs to protect itself (*11*).

The US Naval Base at Yerba Buena Island in San Francisco Bay (*12*) and the mining town of Gunnison, Colorado (*13*), also offer potential lessons for contemporary pandemic influenza preparedness planning. Under the direction of public health officers, the still-healthy island and mountain town essentially cut off all contact with the outside world to shield themselves from the incursion of influenza. The 2 sites saw almost no cases of infection and thus experienced no deaths, for 2 and 4 months, respectively.

Most important, these communities enacted a policy we have termed protective sequestration, or the measures taken by the authorities to protect a defined and still-healthy population from infection before it reaches that population. These measures include the following: 1) prohibitions on members of the community from leaving the site; 2) prohibitions against visitors from entering a circumscribed perimeter; 3) typically placing in quarantine those visitors who are allowed to enter for a period of time before admission; and 4) if available, taking advantage of geographic barriers, such as an island or remote location.

**Figure 1:**
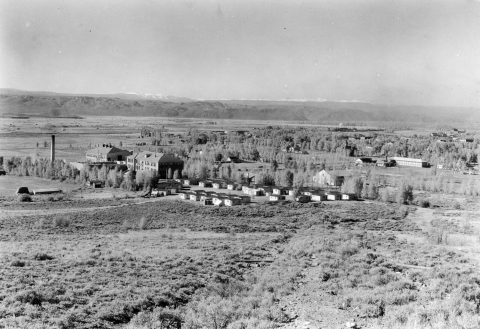
Western State College, Gunnison, Colorado. Source: Denver Public Library, Western History Collection, call no. X-9302.

**Figure 2:**
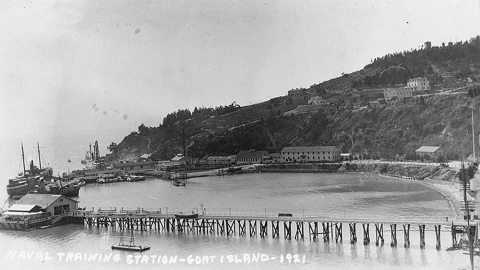
Naval Training Station, San Francisco, California. View looking south over the wharf area, from the eastern end of Yerba Buena ("Goat") Island, 1921. Long Wharf is in the foreground, lined with rowing boats on davits. Beyond is Navy Wharf, with the receiving ship Boston (1887–1946) at far left. The Lighthouse Wharf is beyond that. Collection of Eugene R. O'Brien. Photo #NH 100361. US Naval Historical Center photograph. From US Naval Historical Center (available from http://www.history.navy.mil/photos/images/i00000/i00361.jpg).

Several themes emerged from our historical research. First, coordination among public agencies is essential to any effective public health response. Despite some tension among city, county, and state officials in Gunnison, their relatively smooth cooperation may have played a role in their implementing and maintaining strict public health measures. Second, neither Gunnison nor Yerba Buena could have escaped the flu without full cooperation from the local population. Gunnison's low population density and self-sufficient ranching lifestyle made it easier for residents to bide their time (Figure 1). At Yerba Buena, the military chain of command mandated the cooperation of sailors and allowed the island's commander to close off the base from the outside world with little interference (Figure 2). Finally, these communities had the advantage of early warnings to prepare their populations. Both tracked influenza's westward movement from August to September and, unlike communities along the East Coast, could implement protective health strategies before cases appeared at their doorsteps.

One would like to think that the 6 communities we identified fared better than others because of the NPIs they enacted. While we cannot prove this for any of them, the case is perhaps strongest for Yerba Buena and possibly Gunnison. Further complicating our task, in addition to the uneven quality and quantity of information available for study, is that some of these communities were sparsely populated and geographically isolated, and all of them were subject to the vagaries of how the influenza virus affected populations. Indeed, these communities represent the exception rather than the rule in terms of how most American communities experienced the influenza pandemic of 1918–1920 (*14**,**15*). This leads to several intriguing questions regarding what these escape communities can teach us about pandemic preparations today, let alone the question of whether such measures can even be replicated.

## Conclusions

First, protective sequestration, if enacted early enough in the pandemic, crafted so as to encourage the compliance of the population involved, and continued for the lengthy time period in which the area is at risk, stands the best chance of guarding against infection. Second, available data from the second wave of the 1918–1920 influenza pandemic fail to show that any other NPI (apart from protective sequestration) was, or was not, effective in preventing the spread of the virus. Despite implementing several NPIs, most communities sustained considerable illness and death. We could not assess how the timing of NPI implementation across the nation affected disease mitigation efforts nor whether these NPIs lessened what might have been even higher rates had these measures not been in place in various locations. Moreover, we could not locate any consistent, reliable data supporting the conclusion that face masks, as available and as worn during the 1918–1920 influenza pandemic, conferred any protection to the populations that wore them (*16*). In fact, evidence suggests that in most American communities NPIs did not prevent the spread of virus in 1918. What remains unclear is the extent to which they may have been partially effective in reducing spread or mitigating community impact.

However inconclusive the data from 1918 are, the collective experiences of American communities from the pandemic are noteworthy, especially in light of the fact that, if faced with a pandemic today, we would likely rely on many of these same NPIs to attempt to mitigate the spread of the infection until pharmacological supplies of vaccine and antiviral agents were available (*17**–**19*). It is true that the United States of today is a much different nation than it was in 1918, with a larger, more mobile, and more complex society. It is equally true that the communities we examined were all small and relatively isolated (or isolatable). Nevertheless, in the event of another influenza pandemic, many specific subcommunities (e.g., military installations, college and university campuses, nursing homes) may wish to consider protective sequestration measures as potential means to prevent or delay the onset of epidemic influenza in their populations.
